# Influence of seasonal variation on reported filarial attacks among people living with lymphedema in Ghana

**DOI:** 10.1186/s12879-019-4084-2

**Published:** 2019-05-20

**Authors:** Alexander Kwarteng, Yarhands Dissou Arthur, John Kanyiri Yamba, Augustina A. Sylverken, Priscilla Kini, Samuel Terkper Ahuno, Ellis Owusu-Dabo

**Affiliations:** 1 Department of Biochemistry and Biotechnology, Kwame Nkrumah University of Science and Technology, PMB, KNUST, Kumasi, Ghana; 2 Kumasi Centre for Collaborative Research in Tropical Medicine, Kwame Nkrumah University of Science and Technology, KNUST, Kumasi, Ghana; 3 Department of Interdisciplinary Studies, University of Education Winneba, Kumasi Campus, Kumasi, Ghana; 4 College of Agriculture Education, University of Education Winneba, Asante Mampong, Mampong, Ghana; 5 Department of Theoretical and Applied Biology, Kwame Nkrumah University of Science and Technology, KNUST, Kumasi, Ghana; 6 Department of Global and International Health, School of Public Health, Kwame Nkrumah University of Science and Technology, KNUST, Kumasi, Ghana

**Keywords:** Lymphatic filariasis, Lymphedema, Adenolymphangitis, Ghana

## Abstract

**Background:**

Lymphatic Filariasis (LF) is a vector-borne neglected tropical disease caused by the filarial nematode parasites that can lead to the disfiguring swelling of the limbs (lymphedema or elephantiasis for late stage) and/or genitalia (hydrocele) in men. Growing evidence suggests that not only are filarial lymphedema patients confronted with huge societal stigma and discrimination, but also experience acute filarial attacks accompanied by swelling of the affected part(s), fever, wounds and peeling of the skin of affected limbs(s). However, the extent to which seasonal variation influence filarial attacks among people with lymphedema was highly speculated without empirical evidence and was thus investigated.

**Methods:**

In light of this, a cross-sectional study where 142 (70.4% females and 29.6% males) lymphedema patients were recruited from 8 established Wuchereria bancrofti endemic communities in the Ahanta West District, Ghana was carried out to investigate the prevalence and seasonal variation (rainy/wet and dry seasons) of acute filarial attacks. Chi-square test was used to test for association between frequency of attacks and seasonality. The STROBE guidelines for reporting cross-sectional studies was adopted.

**Results:**

The average lymphedema leg stage was 2.37 and 2.33 for left and right legs, respectively, while mossy lesions, sores and ulcers were observed among 33.1% of patients with late stage disease (elephantiasis). It was found that 97 (68.3%) of the study participants experience filarial attacks during the wet season and 36 (25.4%) reported the incidence of filarial attacks during both seasons (wet and dry) while 9 (6.3%) of the study participants did not experience any attack at all.

**Conclusions:**

Findings from the present study show compelling evidence that the frequency and the prevalence of filarial attacks is significantly increased during wet seasons compared to the dry season.

**Electronic supplementary material:**

The online version of this article (10.1186/s12879-019-4084-2) contains supplementary material, which is available to authorized users.

## Background

Lymphatic filariasis (LF), the arthropod-borne disease, is a significant public health problem in most developing countries [1]. The disease affects more than 120 million people worldwide and is transmitted by thread-like nematode parasites including *Wuchereria bancrofti* which accounts for 90% of the infection [2]. This disease manifests overtly as lymphedema (LE) and hydrocele. It is worth noting that although transmission can be targeted with existing microfilaricidal drugs, such as Diethylcarbamazine, Ivermectin, and Abendazole, morbidity associated with LF (lymphedema and hydrocele) which affects > 40 million people, cannot be effectively treated by anti-filarial chemotherapy without the complementary activity of the host immune response [3]. People with filarial pathologies (lymphedema and hydrocele) frequently present with oedema in the affected parts with repeated attacks of adenolymphangitis (characterised by lymph node swelling, excruciating pains and feverish conditions) which confines most patients to bed preventing them from engaging in social and economic activities [4, 5]. On the other hand, not only does LF lead to disfiguring pathologies and other complications but may result in depressive mental illness due to the social stigmatization against infected individuals [6].

Acute filarial attacks are an important issue for lymphedema patients, therefore better understanding of them and their triggers are required. Rao et al. used a prospective study to investigate the frequency, nature and course of acute filarial attacks in a *Brugia malayi* endemic setting and documented higher incidence during the rainy season in Kerala [7]. Pani et al reported that the mean number of ADL episodes for a year was 4.2 and increased with grade of the disease but was independent of age and grade [8].

Filarial attacks is believed to be orchestrated by several factors including the release of the endosymbiont *Wolbachia* in affected host [9], release of antigens after death of adult worms, bacterial and fungal infections in already compromised lymphatic vessels and infective bites from mosquitos (which is significantly influenced by rainfall patterns). There is sufficient evidence that mosquito vector density increases during rainfall [10, 11], however, as to whether the increased vector density during raining season is associated with acute filarial attacks remains to be fully established. An understanding of seasonal variations of filarial attacks among lymphedema patients is key in the development of interventions at specific time points for effective management of lymphedema. In light of this, we sought to determine the seasonal variation of acute filarial attacks in some selected LF (*W. bancrofti*) endemic communities in Ghana.

## Methods

### Study design

In this study, a cross-sectional design was used and the prevalence and seasonal variation (wet and dry season) of acute attacks were measured retrospectively. Lymphedema patients were recruited from 8 communities in Ahanta West District of Western Region, Ghana known to be endemic for lymphatic filariasis (predominantly *W. bancrofti*). Structured questionnaires (Attached as supplementary file) were used to collect information on incidence and frequency of acute filarial attack over the past year from lymphedema patients. Patient recruitment was carried out for two weeks in July, 2018.

### Study area

**Fig. 1:**
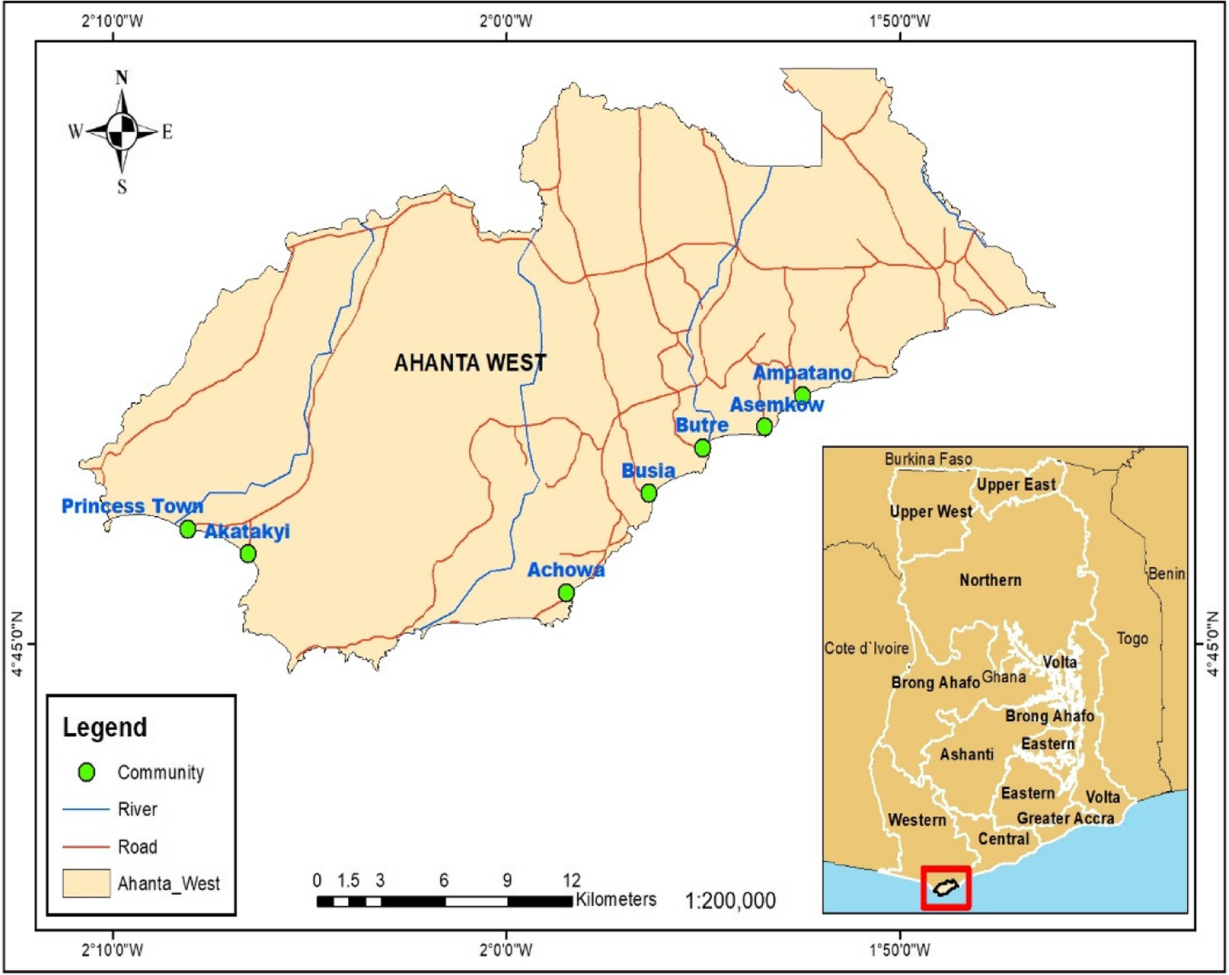
Map of study area. The figure shows the map of the study area (Ahanta West District). The eight [8] participating communities were indicated in green dots

The study was conducted in 8 filarial endemic communities (Achowa, Dixcove, Ampatano, Asemkow, Butre, Busua, Akatakyi and Princess Town) in the Ahanta West District of the Western Region, Ghana (Fig. 1). Dunyo et al previously reported that these communities were hyperendemic for *W. bancrofti* with microfilariae prevalence ranging from 5 to 20% in average of 500–800 of the population (Dunyo et al.*,* 1996), however, the prevalence of lymphedema awaits to be determined. These communities lie along the coast and are hyper-endemic for filarial infections.

The Ahanta West district has a population of 106,000 with economic activities such as fishing, farming, trading among others. While the communities in the Ahanta West district are large and closer to each other; they are mainly linked with third class roads, which makes travelling difficult especially in wet seasons. Mass drug administration for the control of human filarial infection had begun in these communities for the past 23 years. Of note, doxycycline treatment was carried out in a previous study [12] for all the communities but for Akatakyi and Princess Town.

### The average rainfall and humidity

The Ahanta West district is located within the western equatorial climatic zone of Ghana, with the highest mean temperature of 34 °C, recorded between March and April, while the lowest mean temperature of 20 °C is experienced in August. It is interesting to note that the district records a high relative humidity ranging between 75 to 85% in the rainy season and 70 to 80% in the dry season. The district is located in the wettest region of Ghana. It experiences a double maxima rainfall of over 1700 mm. The rainy/wet season is between the months of April and September, with the greatest volume recorded between April and July.

### Patient recruitment and data collection

The study was approved (CHRPE/AP/575/18) by the Committee of Human Research and Publications and Ethics, School of Medical Sciences, Kwame Nkrumah University of Science and Technology, KNUST, Kumasi, Ghana. Approval was also sought from District Health Directorate at Agona Nkwanta in the Ahanta West District, Ghana. With support from the Disease Control Unit at the Dixcove District Hospital, endemic communities within the Ahanta West District were identified from which 8 communities were randomly selected. Information about the study was sent out using public address systems in the various communities for patients to converge at a specific place in each community.

Study participants were approached by research assistants and had the study protocols read out and explained to them in their local dialects after which consent was sought and documented either by signing or thumb printing informed consent forms. To be included in the study one had to be resident in the community for more than 5 years, with lymphedema, be willing to take part in the study and should be 18 years and above. Any person with reported evidence of lymphadenitis, lymphangitis, lymphedema and painful tender swelling of scrotum in male was considered to have an acute filarial attack. However, increase in swelling of any of the body part without evidence of fever, pain and tenderness was not considered as acute filarial attack. Cases did not include those with lymphedema of the breast. For operational definition of seasonal variation, there are 2 major seasons in Ghana which are the rainy and dry seasons. The rainy season begins in April and ends in August whereas the dry season begins in September and ends in March.

All those who did not meet the inclusion criteria (non-lymphedema individuals, mental health) were excluded. Lymphedema leg staging were carried out by experienced research scientists with support from community health workers and officials from the Disease Control Unit, Dixcove District Hospital. Incidence of acute filarial attack, seasonal variation, and duration of attack were all assessed using structured questionnaires (Additional file 1).

### Clinical grading of lymphedema

Grading of lymphedema was based on physical signs on affected limbs using WHO seven-stage system. Grade I: Pitting edema of the limb that is reversible overnight. Stage I lymphedema patients rarely has acute attacks, entry lesions, or a bad odour.

Grade II: Pitting or non-pitting edema that is not reversible overnight without lymphedema management and the skin is normal. Patients with stage 2 lymphedema may have entry lesions, or mild bad odour and experience acute attacks.

Grade III: Presence of one or more shallow skin folds. Entry lesions between the toes and bad odour are more common than in stage 2. Stage III lymphedema patients may have occasional acute attacks.

Grade IV: Presence of knobs (bumps, lumps, or protrusions) on the skin. Patients with stage 4 lymphedema experience occasional acute attacks and will have entry lesions between the toes and a bad odour.

Grade V: The main feature is the presence of deep skin folds and lymphedema can extend above the knee. Most patients have entry lesions between the toes and/or folds and a bad odour and experience occasional to frequent acute attacks.

Grade VI: The presence of mossy lesions is the main feature of stage VI. On the surface of the foot (especially the toes), very small elongated or rounded small knobs can be clustered together, giving rise to the peculiar appearance of “mossy foot”. Patients frequently present with wounds in the skin.

Grade VII: Stage VII lymphedema patients are unable to adequately or independently perform routine daily activities such as walking, bathing, cooking, etc. They have frequent acute attacks and large legs or arms with deep folds. They always have entry lesions between the toes and skin folds. Bad odour is strong. Wounds in the skin are commonly present, and lymphedema extends above the knee in most patients.

### Statistical analysis

Socio-demographic information was presented as frequencies. Chi square test was used to compare stage of lymphedema on left and right legs among lymphatic filariasis patients. Association between the season (wet/rainy and dry season) and frequency of LF attacks per year was determined using Chi square test after the latter was recoded into categorical variable (ie < 1, 1–2, 3–4, 5+). We determined statistical significance at *p* < 0.05 level.

## Results

### Study population characteristics

In total, 142 lymphedema patients were recruited from 8 LF endemic communities in the Ahanta West District, Ghana. There were more females (70.4%) than males (29.6%) as shown in Table 1. The median ± SD age of the study participant was 52.0 ± 15.6 years. Majority of the study participants were either farmers (28.9%) or fish mongers (20.4%), whereas majority of the remaining were involved in menial jobs/petty trading. Of concern, a significant number of the study participants were unemployed (21.1%). The average duration (years) ± SD of lymphedema was 15.5 ± 12.5 years. Patients with lymphedema had leg stages ranging from stage 1 to stage 7, with an average leg stage of 2.37 and 2.33 for left and right legs, respectively (Table 2). Mossy lesions, sores and ulcers were observed among 33.1% of patients with late stage disease (elephantiasis).

**Table 1:** Proportion of LF patients requited from the study communities

Community	Number recruited (%)	Male/Female
Dixcove	21 (14.8%)	11/10
Achowa	5 (3.5%)	1/4
Busua	15 (10.6%)	3/12
Butre	18 (12.7%)	5/13
Ampatano	15 (10.6%)	4/11
Asemkwo	20 (14.1%)	3/17
Princes Town	18 (12.7%)	4/14
Akatakyi	30 (21.1%)	10/20
Total	142	42/100

The table shows the proportion of LF patients recruited from the eight [8] participating communities (Dixcove; Achowa; Busua; Butre; Ampatano; Asemkwo; Princes Town; Akatakyi) from the Ahanta West District, Ghana

**Table 2:** Grade of lymphedema among lymphatic filariasis patients

Grade	Left leg n (%)	Right leg *n* (%)	Chi square test (*p* value)
No obvious signs	38 (26.8)	38 (27)	0.001
1	3 (2.1)	1 (0.7)	
2	39 (27.5)	37 (26.2)	
3	30 (21.1)	31 (22)	
4	12 (8.5)	18 (12.8)	
5	6 (4.2)	8 (5.7)	
6	12 (8.5)	7 (5)	
7	2 (1.4)	1 (0.7)	

The table shows the grade of lymphedema on lymphatic filariasis patients’ left and right feet. Majority of LF patients are stage 3 on both left and right legs, respectively (27.5 and 26.2%). Chi-square test of independence indicates that there was an association between grade of lymphedema on left and right legs (*p* < 0.001)

### Prevalence and frequency of filarial attacks among lymphedema patients

In this study, we found that out of 142 study participants, 133 (94%) experienced filarial attacks the previous year (Table 3). More women reported that they suffer from filarial attacks than men however the reported incidence of acute filarial attacks between gender was not statistically significant (*p* = 0.058). In addition, the mean (±SD) acute filarial attacks per year was 3.2 (±2.6).

**Table 3:** Incidence of filarial attacks among study participants

	Gender			
Do you get attacks?	Female *n* (%)	Male *n* (%)	Total	Fischer exact test (*P*- value)
Yes	91 (91%)	42 (100%)	133 (93.7%)	0.058
No	9 (9%)	0 (0%)	9 (6.3%)	
Total	100 (100%)	42 (100%)	142 (100%)	

The table above shows the reported incidence of filarial attacks among male and female lymphedema patients. Generally more females reported attacks. However, the reported incidence of attacks between male and female respondents was statistically independent as shown by the Fischer exact test (p = 0.058)

Having determined the prevalence of filarial attacks among the study participants, we next investigated the frequency of attacks per year among these individuals. We observed that out of the 142 participants, 30 (21.1%) had at least 2 attacks per year while 28 (19.7%) had at least an attack per year. Of note, 1 patient reported of 14 (0.7%) attacks per year (Table 3).

### Filarial attacks during the seasons among people living with filarial lymphedema

It was found that 97 (68.3%) of the study participants experience attacks during the wet season, 36 (25.4%) reported the incidence of filarial attacks during both seasons (wet and dry), while this was not applicable in 9 (6.3%) of the study participants, given that they have no attacks at all (Table 3).

Given that filarial attack appears to be influenced by seasonal changes, we determined whether there was any association between frequency of LF attacks and seasonal variation on LF attacks. To determine the prevalence of filarial attacks, structured questionnaires were adopted and respondents were made to self-report the incidence and seasonal variations of acute filarial attacks. For some, 9 (6.3%) of the study participants, filarial attacks were not common while 133 (93.7%) reported the incidence of acute filarial attacks with varying severity and frequencies (Table 2). Following a chi-square analysis (x^2^ = 143.8, *p* value = 0.00, Table 4), we showed an association between season of attacks and frequency of attacks.

**Table 4:** Influence of Season on Frequency of LF Attacks

Influence of Season on Frequency of LF Attacks								
	Frequency of Attacks							
		< 1	1–2	3–4	5+	Total	Chi-square	*P*-value
Season of Attacks	N/A	9	0	0	0	9	143.8	0.00
	Wet	0	39	34	24	97		
	Wet and Dry	0	19	10	7	36		
Total	Total	9	58	44	31	142		

The table shows a cross tabulation between season of filarial attacks (i.e., wet and dry seasons) and frequency of attacks. Chi-square test of independence (χ^2^ = 143.8, *p*-value < 0.00) was shows there is a significant association between the two variables

## Discussion

Filarial attacks present huge economic burden to affected individuals [13]. The main control program in LF endemic regions focuses primarily on mass drug administration (MDA) with little support for people with the morbidity [14]. In this study, we found a higher frequency of filarial attacks among LE patients. Although filarial attack is common among LE patients, it was quite alarming to observe over 94% of the study participants reporting of it. Similarly, studies conducted in India and Tanzania also reported high incidence of attacks among LE individuals [8, 15]. In our study more women reported that they suffer from filarial attacks than men however the reported incidence of acute filarial attacks between gender was not statistically significant (*p* = 0.058). This was however in contrast to the study conducted in Tanzania where more males experienced acute attacks than females [15]. In contrast Gyapong and colleagues’ field observation in Ghana where females had higher prevalence of acute filarial attacks was in agreement with that of the findings in the present study [16].

Interestingly, the prevalence of filarial attacks in the Ahanta West district reported by the present study appears to be much higher after 20 years of MDA intervention in the study communities. This findings corroborate that of Gyapong et al who determined the epidemiology of acute adenolymphangitis due to lymphatic filariasis in northern Ghana [16] and found high prevalence of filarial attacks among LF patients.

The Ahanta West District is one of the most highly endemic districts for lymphatic filariasis in Ghana [17]. Despite over 20 years of MDA in the district, the number of cases continue to increase with new cases recorded yearly [18]. What accounts for high prevalence of filarial attacks is unknown but could be linked with the extent of the endemicity of the infection in the study communities as well as the filarial species responsible for ongoing transmission of LF in the communities. Rao and colleagues suggested that the species of filarial nematodes in ongoing transmission may account for the dynamics and severity of acute filarial attacks after comparing their findings conducted in a *B. malayi* setting with those conducted in *Brugia timori* and *bancrofti* endemic area [7]. To add to that, it is suggested that poor compliance to mass drug administration programs is one of the main causes which results in increased infection intensity [19].

Another plausible reason which could account for our observation is the fact that, majority of the LE patients in the study communities were not aware of foot-care hygiene practices. Even where some of the study participants were aware, there was little evidence to show if they complied with such practices. Generally, foot care hygiene is believed to significantly reduce filarial attacks [20] through reduction of microbial population on affected limbs, i.e. bacteria and fungi, which are known to complicate human filarial pathologies [20, 21]. However, it would be interesting to investigate factors underlying seasonal variations in foot hygiene (i.e. patients ability to carry out foot hygiene at specific times in the year), through larger epidemiological studies to be able to develop interventions for promoting foot hygiene at specific times in the year and in turn mitigate seasonal variations in acute filarial attacks.

In this study, the frequency of filarial attacks among the study participants was high. Here, we found that majority of the study participants had at least 2 attacks per year, while others reported more counts of attacks per year. A previous study conducted in some communities in Ahanta West District reported that LE attacks ranged from one to ten per year [22].

Although the factors influencing the high frequency of attacks among the study population are not clear, one has to take into consideration individual personal hygiene, stage of lymphedema, presence of wounds, as well as occupational hazards. It is also important to establish that participation in MDA programs could help explain this observation. Most of the study participants reported not to take part in the MDA because they did not observe much improvement in the leg stage after several years of participation. While these factors could come close to explaining our observation, we also admit that other unknown factors may account for the high frequency of attacks in the study population.

One of the key findings made in this study is the account of higher frequencies of attacks recorded during the wet season. However, this was not uncommon given that filarial attacks are known to be influenced by environmental conditions [9]. A previous study conducted in Northern Ghana, which is almost 900 km from the current study site had reported similar observation [16]. Our study was conducted in Ahanta West District which lie within the wettest region in Ghana. However, why filarial attacks in majority of the study participants coincide with wet (rainy) seasons remains to be addressed.

Wet seasons are characterised by a drop-in temperature, which may lead to the onset of filarial attacks. The average temperature recorded during the wet season within the district was 20 °C with heavy rainfall. However, acute filarial attack may be mediated by infective bites from mosquitos (which is significantly influenced by rainfall patterns). Therefore the increase in malaria vector density especially during rainfall [10, 11] may predispose LE patients to infective mosquito bites which may facilitate the incidence and frequency of filarial attacks in endemic Ghanaian communities. Similarly, this scenario is akin to arthritis, a condition which is also highly influenced by changes in atmospheric temperatures [23, 24]. As atmospheric pressure falls, tissues in the body may expand. As the tissues expand, they put more pressure on nerves that control pain signals [24]. In this study, we have found compelling evidence that incidence of filarial attack associates strongly with rainy season compared to dry season.

## Conclusion

In this study we determined the frequency and seasonal variations of acute filarial attacks among LE patients in LF-endemic communities in Ahanta West District in Ghana. We observed that out of the 142 participants, 30 (21.1%) had at least 2 attacks per year, 28 (19.7%) had at least an attack per year. Furthermore, we observed an association between season of attacks and frequency of attacks supporting previous research of seasonal variability of acute filarial attacks. Findings from this study has huge implication for people living with LE given that most of them are involved in farming activities. Thus, the high frequency of attacks during the raining seasons may significantly impact productivity and possibly increase the economic burden of people living with filarial lymphedema. Although, our study underscores the challenge of acute attacks in the management of LF in Ghana, we strongly believe that further or large prospective studies on the subjects may provide more insights with regards to the frequency, nature and triggers of acute filarial attacks which can be used as basis to change management practices and health policy formulation.

## Additional file

Additional file 1: Study questionnaires. The reported incidence of filarial attacks among lymphedema patients in the study communities were assessed using the structured questionnaire. The questionnaire was based on authors’ own constructs. (DOCX 93 kb)

## Abbreviations

LE: Lymphedema
LF: Lymphatic filariasis
MDA: Mass drug administration
