# Supplementary material for: Influence of seasonal variation on reported filarial attacks among people living with lymphedema in Ghana
Source: BMC Infect Dis. 2019 May 20;19:442. doi: 10.1186/s12879-019-4084-2 (PMC6528377; doi:10.1186/s12879-019-4084-2)
Supplement: Supplementary file 1 — Study questionnaires. The reported incidence of filarial attacks among lymphedema patients in the study communities were assessed using the structured questionnaire. The questionnaire was based on authors’ own constructs. (DOCX 93 kb) [file 12879_2019_4084_MOESM1_ESM.docx]

Title: The frequency of filarial attacks among lymphedema patients: A focus on leg stage, adherence to foot-care hygiene, and the impact of seasonal variations.

Name of interviewer……………………………………… Date: ……………………..

|  |
| --- |

Indiv. No.: - Village:

Sex: M F

Age: Weight: kg Date:

**Study specific history**

Previous treatment: Doxycycline............................................………………………...

Ivermectin / albendazole: Last intake (mm/yy)

No. of rounds:

If yes, please specify:…………………………………..

Other drugs (antibiotics, pain killer)..............................................

Lymphedema (LE): yes no since: years

If yes, please specify:

Treatment in case of any infection: yes no

If yes, please specify:

LE staging: Left Leg…………………………………………

Right Leg…………………………………………..

How long have you had this condition? ........................................months/years

Wound on affected leg(s)? yes no

Has the wound on the affected leg ever been treated? yes no

If yes, what type of treatment ……………Antibiotics/ Traditional

Has swab(s) been taken from wound? yes no

Has swab(s) been taken from toes of affected leg(s) yes no

**Frequency of attacks**

Do you have attacks? yes no

State the number attacks per month………………..

State the number of attacks per year……………….

What is the duration (i.e. number of days) for a typical attack? ………….

When is the attack more? Rainy season Dry season

Does the attack results in peeling of the leg (affected part)? yes no

Do you get access to health care during attacks? yes no

Do you report attacks to hospitals? yes no

Are you given any intervention when you report attacks to the hospitals? yes no

Which of the following would you want to visit during attacks? Hospitals Native/Traditional doctor if other, specify ……………………….

Do you usually raise or exercise the affected legs in the course of the day? yes no

**Social history**

Patient lives in the village since years

Patient is able to carry out work: yes no

Has this condition made you change jobs yes no

Specify work the patient is carrying out: …………………………

Do you usually wash your affected feet? yes no

How many times do you wash your affected leg in a day? ………….. times

What do you wash your feet with? Soap and water ordinary water without soap

What is the source of water used for washing your feet? well pipe river/stream

How close is your water source to your house? ……….. meters/kilometre

Can you afford soap and detergents? yes no

If No, who provides? Family members Friends NGO

Do you benefit from NGO sponsored leg washing programmes? yes no

**Criteria for Study Enrolment**

|  | **YES** | **NO** |
| --- | --- | --- |
| 1. Male or female aged between 18 – 70 years old. |  |  |
| 1. Lymphedema of any of the legs |  |  |
| 1. Lymphedema of the arm |  |  |
| 1. Lymphedema of the breast |  |  |
| 1. Willingness to participate in the study as evidenced by the signing or thumb-printing of the informed consent document. |  |  |
| **Study Inclusion** |  |  |

Interviewer signature …………………………… Date…………………….
